# Prevalence of ultrasonographic gastrointestinal wall changes in dogs with acute pancreatitis: A retrospective study (2012‐2020)

**DOI:** 10.1111/jvim.16414

**Published:** 2022-03-23

**Authors:** Joshua J. Hardwick, Elizabeth J. Reeve, Melanie J. Hezzell, Jenny A. Reeve

**Affiliations:** ^1^ Bristol Veterinary School University of Bristol North Somerset United Kingdom; ^2^ Langford Vets Small Animal Referral Hospital Langford United Kingdom; ^3^ Highcroft Veterinary Referrals Bristol United Kingdom

**Keywords:** canine, gastroenterology, gastrointestinal ultrasound, hepatobiliary

## Abstract

**Background:**

Ultrasonographic gastrointestinal wall changes in dogs with acute pancreatitis (AP) are not well characterized in the literature. No detailed studies have described their prevalence, characteristics, distribution, or clinical relevance.

**Hypothesis/Objectives:**

Describe the prevalence of ultrasonographic gastrointestinal wall changes in a population of dogs with AP and evaluate for associations between the presence of gastrointestinal wall changes and clinical or clinicopathological variables.

**Animals:**

Referral population of 66 client‐owned dogs with AP.

**Methods:**

Retrospective search of clinical records to identify dogs with AP. Clinical variables, clinicopathological variables and ultrasonographic findings were reported using descriptive statistics. A binary logistic regression model was used to evaluate for associations between the presence of gastrointestinal wall changes and clinical or clinicopathological variables.

**Results:**

Sixty‐six dogs were included. Forty‐seven percent of dogs (95% confidence interval [CI], 35.0%‐59.0%; n = 31) with AP had ultrasonographic gastrointestinal wall changes. Gastrointestinal wall changes were most common in the duodenum and identified in 71% (n = 22) of affected dogs. Of dogs with gastrointestinal wall changes, 74.2% (n = 23) had wall thickening, 61.3% (n = 19) had abnormal wall layering, and 35.5% (n = 11) had wall corrugation. In the multivariable model, only heart rate remained an independent predictor of ultrasonographic gastrointestinal wall changes (*P =* .02).

**Conclusions and Clinical Importance:**

Ultrasonographic gastrointestinal wall changes in this population of dogs with AP were common. Increased heart rate was the only independent predictor of gastrointestinal wall changes, which might imply more severe disease. Additional studies are required to elucidate whether ultrasonographic gastrointestinal wall changes reflect disease severity in AP.

**Abbreviations**
ACVIM: American College of Veterinary Internal Medicine
AKI: acute kidney injury
ALP: alkaline phosphatase
ALT: alanine aminotransferase
ANP: acute necrotizing pancreatitis
AP: acute pancreatitis
CBC: complete blood count
cPLI: canine pancreatic lipase
CRP: C‐reactive protein
DKA: diabetic ketoacidosis
GGT: gamma glutamyl transferase
GI: gastrointestinal
IL‐6: interleukin‐6
IMHA: immune‐mediated hemolytic anemia
NSAID: nonsteroidal anti‐inflammatory drug
SRMA: steroid‐responsive meningitis‐arteritis

## 1 INTRODUCTION

Acute pancreatitis (AP) is defined as acute, sterile and generally reversible pancreatic inflammation, characterized histologically by a neutrophilic infiltrate, edema, and necrosis.^1^, ^2^ Antemortem diagnosis typically relies upon a combination of history, physical examination findings, diagnostic imaging (generally ultrasonography or computed tomography), and clinicopathologic findings (hematology, serum biochemistry, and canine pancreatic lipase immunoreactivity). Histopathology is the gold standard for diagnosis of AP, but the morbidity associated with pancreatic biopsy and the heterogeneous distribution of pancreatic inflammation limit its use in practice.^2^ Surrogate noninvasive tests, as described above, are more commonly utilized.

Ultrasonographic features of AP are well described in dogs. Pancreatic enlargement, hypoechoic or heterogeneous pancreatic parenchyma, hyperechoic peripancreatic mesentery, and peripancreatic free fluid are described in naturally‐occurring^3^, ^4^, ^5^, ^6^, ^7^, ^8^ and experimentally‐induced^9^, ^10^, ^11^ pancreatitis. The sensitivity of ultrasound examination for the diagnosis of AP, based upon criteria of pancreatic hypoechogenicity with mesenteric hyperechogenicity, has been reported to be relatively poor at 68%.^12^ A recent study stratifying cases by the presence of 1, 2, or 3 ultrasonographic criteria from pancreatic enlargement, altered pancreatic echogenicity or hyperechogenicity of the surrounding mesentery reported sensitivities of 89%, 72%, and 42% and specificities of 43%, 69%, and 92%, respectively.^3^

Concurrent ultrasonographic gastrointestinal wall changes in dogs with AP are less widely reported in the literature. Gastric wall thickening,^13^, ^14^, ^15^ increased small intestinal wall thickness,^11^, ^15^, ^16^, ^17^ small intestinal corrugation,^5^, ^15^ and blurred or apparent loss of gastrointestinal wall layering^13^, ^14^, ^16^, ^17^ have been reported. A recent case series reported gastric wall changes in 14 dogs with naturally‐occurring AP, suspected to be reflective of gastric wall edema, but no prevalence data were reported.^14^ The prevalence of gastrointestinal wall changes in naturally‐occurring AP has not previously been well characterized. Although no mechanistic explanation for gastrointestinal wall changes in AP has been determined, it is reasonable that the anatomic proximity of the pancreas to the pylorus, duodenum, transverse colon, and jejunum could result in extension of local inflammation to these structures.

Our aims were (i) to report the prevalence and nature of ultrasonographic gastrointestinal wall changes in a referral population of dogs with AP, and (ii) explore associations between the presence of gastrointestinal wall changes and observed clinical and clinicopathologic variables. We hypothesized that the presence of neutrophilia, left shift, or toxic neutrophils, as surrogate markers of increased severity of inflammation, would be significantly associated with the presence of ultrasonographic gastrointestinal wall changes.

## 2 MATERIALS AND METHODS

### 2.1 Data collection and case selection

Ours was a retrospective cross‐sectional study. The Small Animal Internal Medicine service case database at Langford Vets, University of Bristol, was searched from February 2012 to August 2020 using the search terms “pancreatitis, pancr*, AP, and ANP” (acute necrotizing pancreatitis) to identify dogs with a diagnosis of AP. For inclusion in the study, cases were required to meet all of the following inclusion criteria:

- A full clinical history available for review, provided by the referring veterinary surgeon.
- Clinical signs compatible with a diagnosis of AP to include at least 2 of: vomiting, diarrhea, abdominal pain, lethargy, hyporexia, or jaundice.
- Abnormal canine pancreatic lipase assay (Spec cPLI, Idexx Laboratories, Wetherby, United Kingdom) or a cholestatic pattern on serum biochemistry as indicated by a disproportionate increase in serum alkaline phosphatase (ALP) or gamma glutamyl transferase (GGT) activities with respect to alanine aminotransferase (ALT) activity.
- At least 1 ultrasonographic abnormality compatible with a diagnosis of AP from the following criteria: pancreatic enlargement, parenchymal heterogenicity, parenchymal hypoechogenicity, hyperechogenicity of the peripancreatic mesentery or peripancreatic free fluid accumulation.
- An abdominal ultrasound examination with ultrasound report and still or real‐time images available for review.

Cases were excluded based upon the following criteria:

- Evidence of preexisting gastrointestinal disease in the dog's history characterized by ≥2 episodes of vomiting or diarrhea in the 2 months before presentation, excluding clinical signs considered related to the current problem.
- A laparotomy performed in the 2 weeks before referral.
- Administration of corticosteroids or nonsteroidal anti‐inflammatory drugs (NSAIDs) in the month before presentation.
- Suspicion of pancreatic neoplasia clinically or ultrasonographically.
- Any concurrent disease process that might have altered ultrasonographic gastrointestinal wall appearance.

For evaluation of associations between clinicopathological variables and the presence of gastrointestinal wall changes, cases were further excluded if hematological data was lacking or if diagnostic evaluations identified any concurrent disease, other than AP, that might have resulted in a proinflammatory state or affect other clinicopathologic variables.

### 2.2 Patient characteristics

Patient signalment, body weight, body condition score, clinical signs, and physical examination findings were extracted from the medical records. Patient comorbidities were recorded where present.

### 2.3 Ultrasonographic examinations

All ultrasonographic examinations were either performed or supervised by a board‐certified radiologist using an Acuson S2000 ultrasound system (Siemens GmBH, Erlangen, Germany) before 2019 or an Acuson S3000 ultrasound system (Siemens GmBH, Erlangen, Germany) from 2019 onward. All ultrasonographic images were retrospectively reviewed by a board‐certified radiologist (E.R.) to confirm ultrasonographic evidence of AP and to evaluate for the presence or absence of gastrointestinal wall changes. Changes were then further characterized according to the following criteria:

- Wall layering: reported as either normal or abnormal and specific abnormalities characterized as either an ill‐defined appearance affecting specific layers, an ill‐defined appearance affecting all layers or complete loss of wall layering.
- Wall thickening: reported as either normal or abnormal and specific abnormalities characterized as either generalized thickening or thickening with respect to a specific layer or layers. Reference values for normal gastrointestinal wall thickness were based on published data.^18^
- Intestinal corrugation, reported as absent or present, with anatomic location(s).

### 2.4 Clinicopathologic data

All hematological assays were performed using an Abbott Cell‐Dyn 3700 hematology analyzer (Abbott Laboratories, Illinois, USA) before 2015 and a Siemens Advia 2120 hematology analyzer (Siemens Healthineers, Erlangen, Germany) from 2015 onward, all of which were accompanied by a manual smear examination, performed by, or under the supervision of, a board‐certified clinical pathologist. Reported hematological data included segmented neutrophil count, band neutrophil count and neutrophil morphology. Serum biochemical assays were performed using Thermo Scientific Konelab Prime 60i (Thermo Fisher Scientific, Waltham, MA) for the entire study period. Reported serum biochemical data included ALT, ALP, and GGT activities and total bilirubin concentration. The cPLI assays were performed by IDEXX Laboratories (Wetherby, United Kingdom).

### 2.5 Statistical analysis

Descriptive statistics (median, range) were used to present patient characteristics, clinicopathologic findings, and ultrasonographic findings. Ninety‐five percent confidence intervals (CI) were calculated for percentages using the latest available reported total UK dog population of 8.9 million.^19^ A binary logistic regression model was used to assess for associations between clinical and clinicopathological variables and the presence or absence of ultrasonographic gastrointestinal wall changes. Values of *P <* .05 were considered significant. Variables that reached significance in univariable analysis were carried forward to multivariable analysis. A commercial statistical software package (IBM SPSS Statistics for Windows, v.25.0, IBM Corp, Armonk, New York) was used for all statistical analyses.

## 3 RESULTS

### 3.1 Patient characteristics

**FIGURE 1:**
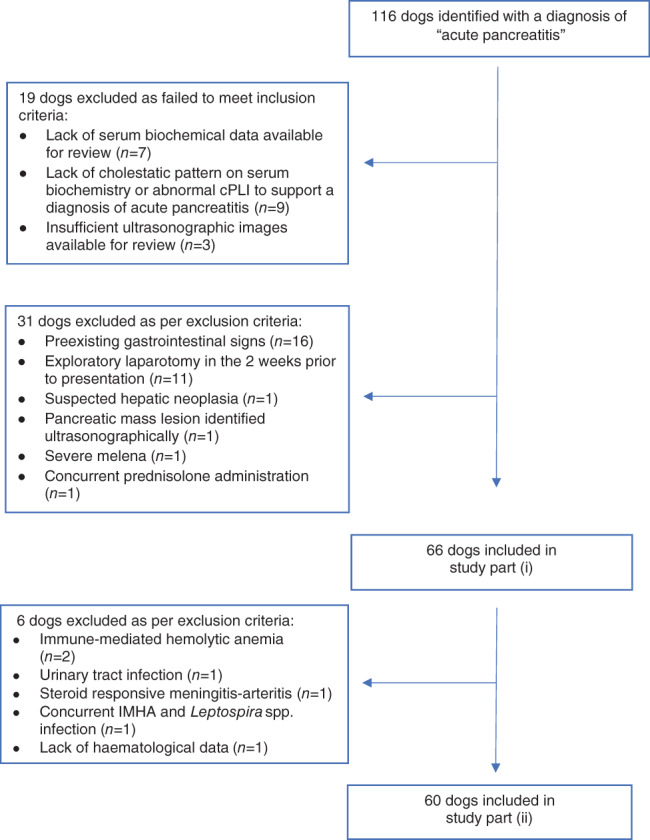
Flowchart demonstrating case exclusion for parts (i) and (ii) of the study. cPLI, canine pancreatic lipase

From the initial search of the case database, 116 dogs were identified as having a diagnosis of AP recorded. Figure 1 demonstrates how 50 dogs were subsequently excluded through failure to meet inclusion criteria (n = 19) or fulfilling exclusion criteria (n = 31). Sixty‐six dogs were included in part (i) of the study. Six dogs subsequently were excluded from part (ii) of the study, either because they lacked hematological data (n = 1) or because their comorbidities were considered likely to confound interpretation of clinicopathologic variables (n = 5). Sixty dogs therefore were included in part (ii) of the study.

Of the 66 dogs included in part (i) of the study, 38 were male (33 neutered and 5 intact) and 28 were female (25 neutered and 3 intact). Twenty‐two breeds were represented in the study as follows: cross breed (n = 11), cocker spaniel (n = 8), Jack Russell terrier (n = 7), miniature schnauzer (n = 6), Yorkshire terrier (n = 5), springer spaniel (n = 4), cavalier King Charles spaniel (n = 3), bichon frise (n = 3), 2 each of patterdale terrier, Labrador retriever, Tibetan terrier, border collie and Siberian husky and 1 each of rottweiler, chihuahua, fox terrier, Norfolk terrier, beagle, Lhasa apso, border terrier, miniature dachshund and West Highland white terrier. Median patient age was 8.3 years (range, 0.4‐12.2 years), median body weight was 11.9 kg (range, 1.4‐39.5 kg) and of the 50 dogs for which body condition score was available, median body condition score was 5/9 (range, 1/9‐8/9).

### 3.2 Clinical signs and physical examination findings

Of the 66 dogs included in part (i) of the study, 92.4% (n = 61) had vomiting reported in their clinical history, 86.4% (n = 57) had lethargy, 75.8% (n = 50) had abdominal pain, 69.7% (n = 46) had hyporexia, and 27.2% (n = 18) had diarrhea. Two, 3, 4, 5, or all 6 of these signs were present in 4.5% (n = 3), 34.8% (n = 23), 33.3% (n = 22), 24.2% (n = 16), and 3.0% (n = 2), respectively. On physical examination, 5 dogs with abdominal pain reported in the history did not have detectable abdominal pain. One‐third of dogs (n = 22) had icteric mucous membranes. Continuous variables identified at physical examination are presented in Table 1.

**TABLE 1:** Continuous variables identified on physical examination

Parameter (units)	n (/66)	Median	Range
Heart rate (beats per minute)	58	120	56‐240
Respiratory rate (breaths per minute)	35^a^	28	12‐64
Rectal temperature (°C)	55	38.5	37.3‐40.1

^a^ An additional 20 dogs were reported as “panting” and 1 reported as “whining” on physical examination which precluded recording of respiratory rates.

### 3.3 Pancreatic ultrasonography

All 66 dogs included in part (i) of the study had ultrasonographic evidence of AP according to the inclusion criteria. The entire pancreas was visualized in 72.7% of dogs (n = 48). For the remaining 18 dogs, the left limb was not visualized in 16, the body not visualized in 1 dog and the left limb and body were not visualized in 1 dog. The right limb was visualized in all dogs.

Regarding pancreatic parenchymal changes, 75.8% (95% CI, 65.5%‐86.1%) of dogs (n = 50) had enlargement of ≥1 pancreatic regions (left limb, right limb, or body). Of those dogs, 82% (n = 41) had enlargement of the right limb, 78% (n = 39) of the pancreatic body, and 68% (n = 34) of the left limb. Almost 73% of dogs (n = 48) had heterogeneous pancreatic parenchyma affecting ≥1 pancreatic regions, and of those 48 dogs, 81.3% (n = 39) had heterogeneity of the pancreatic body, 72.9% (n = 35) of the right limb, and 66.7% (n = 32) of the left limb. Almost 76% of dogs (n = 50) had a hypoechoic appearance to, or hypoechoic regions within, the pancreatic parenchyma affecting ≥1 limbs. Of those 50 dogs, 88% (n = 44) had hypoechogenicity identified within the pancreatic body, 68% (n = 34) within the right limb, and 62% (n = 31) within the left limb.

Regarding changes associated with peripancreatic inflammation, 83.3% (95% CI, 74.3%‐92.3%) of dogs (n = 55) had hyperechogenicity of the peripancreatic mesentery, 10.6% (n = 7) had diffusely hyperechoic mesentery, and 6.1% (n = 4) had no evidence of hyperechoic mesentery. Nearly 38% (n = 25) dogs had localized peripancreatic free fluid, 22.7% (n = 15) had diffuse free abdominal fluid, and 39.4% (n = 26) had no evidence of peripancreatic or diffuse free abdominal fluid.

### 3.4 Gastrointestinal wall changes

Of the 66 dogs included in part (i) of the study, 47% (95% CI, 35.0%‐59.0%; n = 31) had ultrasonographic gastrointestinal wall changes. Of these 31 dogs, 71.0% (n = 22) had wall changes affecting the duodenum, 32.3% (n = 10) the stomach, 16.1% (n = 5) the colon, and 12.9% (n = 4) the jejunum. Overall, gastrointestinal wall changes were focal, affecting only 1 region of the gastrointestinal tract, in 67.7% of dogs (n = 21), and diffuse, affecting ≥1 region of the gastrointestinal tract, in 32.3% (n = 10). Of the 21 dogs with focal changes, 57.1% (n = 12) had duodenal changes, 23.8% (n = 5) colonic changes, 14.3% (n = 3) gastric changes, and 4.8% (n = 1) had jejunal changes. Of the 10 dogs with diffuse changes, 70.0% (n = 7) had gastric and duodenal changes concurrently and 30.0% (n = 3) had duodenal and jejunal changes concurrently.

#### 3.4.1 Abnormal wall layering

Of the 31 dogs with ultrasonographic gastrointestinal wall changes, 61.3% (95% CI, 44.2%‐78.5%; n = 19) had abnormal gastrointestinal wall layering of which the duodenum was affected in 63% (n = 12) of cases, the stomach in 47.4% (n = 9), and the jejunum in 15.8% (n = 3). No dogs had altered colonic wall layering. Of the 12 dogs with abnormal duodenal wall layering, 66.7% (n = 8) had an ill‐defined appearance affecting all duodenal wall layers, 25% (n = 3) had ill‐defined submucosa and muscularis layers and 8.3% (n = 1) had focal complete loss of wall layering. Of the 9 dogs with abnormal gastric wall layering, 33.3% (n = 3) had an ill‐defined appearance affecting all gastric wall layers, 33.3% (n = 3) had an ill‐defined appearance to the mucosa and submucosal layers, 11.1% (n = 1) had an ill‐defined muscularis layer, 11.1% (n = 1) had an ill‐defined submucosal layer and 11.1% (n = 1) had focal complete loss of gastric wall layering. Of the 3 dogs with jejunal wall layering abnormalities, ill‐defined submucosa and muscularis layers were identified in all 3. Of the 19 dogs with abnormal gastrointestinal wall layering, 73.7% (n = 14) had focally abnormal wall layering, and 26.3% (n = 5) had diffusely abnormal wall layering, of which 3 dogs had concurrent gastric and duodenal wall layering abnormalities and 2 dogs had concurrent duodenal and jejunal wall layering abnormalities.

#### 3.4.2 Wall thickening

Of the 31 dogs with ultrasonographic gastrointestinal wall changes, 74.2% (95% CI, 58.8%‐89.6%; n = 23) had gastrointestinal wall thickening of which the duodenum was affected in 70.0% (n = 16) of cases, the stomach in 43.5% (n = 10), the colon in 17.4% (n = 4), and the jejunum in 8.7% (n = 2). Of the 16 dogs with duodenal wall thickening, 81.3% (n = 13) had proportional thickening across all layers and the remaining 18.7% (n = 3) had thickening restricted to the muscularis layer only. Of the 10 dogs with gastric wall thickening, 90% (n = 9) had proportional thickening across all layers and the remaining 10% (n = 1) had thickening restricted to the muscularis and submucosal layers. Colonic wall thickening was proportional across all layers in all dogs (n = 4). Of the 2 dogs with jejunal wall thickening, 50% (n = 1) had proportional thickening across all layers and 50% (n = 1) had thickening restricted to the muscularis layer only. Of the 23 dogs with gastrointestinal wall thickening, 60.9% (n = 14) had focal gastrointestinal wall thickening and 39.1% (n = 9) had diffuse gastrointestinal wall thickening, of which 7 dogs had concurrent gastric and duodenal wall thickening and 2 dogs had concurrent duodenal and jejunal wall thickening.

#### 3.4.3 Wall corrugation

Of the 31 dogs with ultrasonographic gastrointestinal wall changes, 35.5% (95% CI, 18.7%‐52.3%; n = 11) had intestinal corrugation of which the duodenum was affected in 81.8% of cases (n = 9), the jejunum in 18.2% (n = 2), and the colon in 9.1% (n = 1). One dog had concurrent duodenal and jejunal wall corrugation, but corrugation was focal in all other dogs.

#### 3.4.4 Presence of multiple categories of gastrointestinal wall changes

Of the 10 dogs with gastric wall changes, 90% (n = 9) had abnormal layering and thickening concurrently. Of the 22 dogs with duodenal wall changes, 41.0% (n = 9) had abnormal layering and thickening concurrently, 9.1% (n = 2) had concurrent thickening and corrugation and 9.1% (n = 2) had concurrent abnormal wall layering, thickening and corrugation. Of the 4 dogs with jejunal wall changes, 25.0% (n = 1) had abnormal layering and thickening concurrently, 25.0% (n = 1) had abnormal layering and corrugation concurrently, and 25% (n = 1) had wall thickening and corrugation concurrently. No dogs with colonic wall changes had a second concurrent change affecting the colon.

### 3.5 Patient comorbidities

Of the 66 dogs included in parts (i) and (ii) of the study, 37.9% (n = 25) had a concurrent comorbidity. Diabetes mellitus was the most common comorbidity and was identified in 10.6% of the study population (n = 7), followed by diabetic ketoacidosis (DKA) in 9.1% (n = 6) and immune‐mediated hemolytic anemia (IMHA) in 3% (n = 2). Acute kidney injury (AKI), steroid‐responsive meningitis arteritis (SRMA), laryngeal paralysis, American College of Veterinary Internal Medicine (ACVIM) stage B1 myxomatous mitral valve disease, idiopathic epilepsy, hemoabdomen secondary to rodenticide toxicity, and leptospirosis with secondary IMHA were all identified in 1 dog each. Of the 7 dogs with diabetes mellitus, concurrent comorbidities of chronic kidney disease, urinary tract infection, and ACVIM stage B2 mitral valve disease were identified in 1 dog each. The dogs with IMHA, SRMA, urinary tract infection, and leptospirosis were excluded from part (ii) of the study based on the exclusion criteria.

### 3.6 Clinicopathological variables

Clinicopathological variables recorded for the 60 dogs included in part (ii) of the study, that were considered not to have confounding comorbidities, are presented in Table 2. For hematological variables, 76.7% of dogs (n = 46) had neutrophilia, 20% (n = 12) had neutrophil count within the reference interval and 3.3% (n = 2) had neutropenia. Forty‐three percent of dogs (n = 26) had a neutrophilic left shift. 46.7% (n = 28) had toxic change of variable degrees of which 46.4% (n = 13) had 1+ toxicity, 32.1% (n = 9) had 2+ toxicity, 14.3% (n = 4) had 3+ toxicity, and 7.1% (n = 2) had 4+ toxicity. For serum biochemical variables, 78.3% (n = 47), 96.7% (n = 58), and 43.3% (n = 26) had increases in ALT, ALP, GGT activities above the reference interval, respectively, and 63.3% (n = 38) dogs had hyperbilirubinemia. Forty percent of dogs (n = 24) had spec cPL performed, in which 91.7% (n = 22) were abnormal.

**TABLE 2:** Clinicopathological findings from the 60 dogs included in part (ii) of the study

Parameter (units)	n (/60)	Median	Range	Reference interval
Segmented neutrophils (×10^9^)	60	13.43	1.11‐59.45	3.9‐8
Band neutrophils (×10^9^)	26	0.79	0.18‐6.24 × 10^9^	N/A
ALT (U/L)	60	139.5	22‐3608	20‐60
ALP (U/L)	60	968	80‐19 336	0‐110
GGT (U/L)	50	20	0‐345	0‐15
Total bilirubin (mg/dl)	51	0.99	0.2‐25.87	0‐0.58
cPLI (μg/L)	24	907	80‐2000	<200

### 3.7 Association between clinicopathological variables and the presence of gastrointestinal wall changes

In the univariable logistic regression model, as presented in Table 3, both the presence of neutrophil toxic change and heart rate were significantly associated with the presence of ultrasonographic gastrointestinal wall changes. However, when carried forward to the multivariable model, only heart rate remained an independent predictor of the presence of ultrasonographic gastrointestinal wall changes (Table 4).

**TABLE 3:** Univariate binary logistic regression model for associations between clinical and clinicopathological variables and the presence of ultrasonographic gastrointestinal wall changes for the 60 dogs with acute pancreatitis in part (ii) of the study

Variable (number of cases for which parameter available)	*B*	95% CI for *B*	*P* value
Age (n = 60)	0.99	0.819‐1.197	.92
Sex (M vs F) (n = 60)	1.889	0.66‐5.404	.24
BCS (n = 45)	1.082	0.732‐1.599	.69
Weight (n = 59)	0.997	0.915‐1.044	.5
Neutrophilia (n = 60)	0.975	0.291‐3.264	.97
Neutropenia (n = 60)	N/A^a^	N/A^a^	>.99
Left shift (n = 60)	0.153	0.165‐1.328	.15
Toxic change (n = 60)	0.253	0.086‐0.746	**.01**
Heart rate (n = 53)	1.028	1.008‐1.048	**.01**
Respiratory rate (n = 32)	1.026	0.969‐1.086	.37
Temperature (n = 50)	1.123	0.518‐2.435	.77
Abdominal pain (n = 60)	0.581	0.171‐1.975	.38
ALP (n = 60)	1.000	0.999‐1.001	.76
ALT (n = 60)	1.000	1.000‐1.000	.69
GGT (n = 50)	1.001	0.994‐1.008	.76
TBil (n = 51)	1.000	0.994‐1.006	.9

*Note*: *B* refers to the change in predicted log odds of identifying ultrasonographic gastrointestinal wall changes that would be predicted by a 1 unit change in the predictor (eg, the presence of neutrophil toxic change or a 1 beat per minute change in heart rate).

^a^ Only 2 dogs in the study were neutropenic; *B* could therefore not be accurately calculated.

**TABLE 4:** Multivariable binary logistic regression model for associations between clinical and clinicopathological variables and the presence of ultrasonographic gastrointestinal wall changes for the 60 dogs with acute pancreatitis in part (ii) of the study

Variable	*B*	95% CI for *B*	*P* value
Toxic change (n = 60)	0.318	0.008‐1.151	.09
Heart rate (n = 53)	1.024	1.003‐1.044	**.02**

*Note*: *B* refers to the change in predicted log odds of identifying ultrasonographic gastrointestinal wall changes that would be predicted by a 1 unit change in the predictor (eg, the presence of neutrophil toxic change or a 1 beat per minute change in heart rate) with all other variables constant.

## 4 DISCUSSION

We report in detail the prevalence and characteristics of gastrointestinal wall changes in a population of dogs with AP. In terms of patient characteristics, those described here are broadly similar to those reported elsewhere in AP most commonly affects middle‐aged dogs and that crossbreed dogs, miniature schnauzers, cocker spaniels, and Jack Russell terriers appear overrepresented.^12^, ^20^, ^21^, ^22^, ^23^ Reported clinical signs were also similar in that vomiting, lethargy, abdominal pain, hyporexia, and diarrhea were all common in our population of dogs with AP.^3^, ^15^, ^23^ Similar to our findings, hematologic abnormalities reported elsewhere in dogs with AP have included neutrophilic leukocytosis, neutropenia, and left shift. One study reported no association between outcome and hematologic abnormalities in a population of 61 dogs with ultrasonographically or histopathologically confirmed AP,^24^ with another study similarly having reported no relationship between leukocyte counts and duration of hospitalization.^25^ In a population of 70 dogs with fatal AP, 61.7% and 55.4% of dogs had leukocytosis and neutrophilia, respectively, with 55.4% having a left shift. Whereas 3.3% of dogs in that study had leukopenia, none had neutropenia. Similar serum biochemical abnormalities to those identified in our study also have been reported elsewhere, and generally reflect a cholestatic process characterized by increases in ALP and GGT activity disproportionate to ALT activity, sometimes accompanied by hyperbilirubinemia. Increased ALT activity also has been frequently reported, likely as a result of reactive hepatopathy secondary to the cholestatic and local inflammatory process.^12^, ^13^, ^25^, ^26^, ^27^

All of the dogs in our study had ultrasonographic evidence of AP according to the aforementioned criteria, combined with supportive clinicopathological features. Although a cholestatic pattern on serum biochemistry typically has not been necessary for inclusion in previous studies investigating AP, it remains a valuable means by which to increase clinical suspicion for pancreatic pathology, including pancreatitis, and therefore was utilized here. One dog was excluded based upon the presence of a pancreatic mass lesion detected ultrasonographically. It is well recognized that pancreatic mass lesions may be observed ultrasonographically in dogs with AP and in addition to reflecting focal AP, these might alternatively represent pancreatic neoplasia, abscesses, cysts or pseudocysts, although the latter 3 findings, typically would have a fluid filled aspect.^3^, ^10^, ^11^, ^12^, ^28^ Distinguishing between these requires pancreatic histopathology or cytology from fine needle aspirates, neither of which were performed in any of the dogs included in our study.

In our population, 47% of dogs with AP had concurrent gastrointestinal wall changes. Gastrointestinal wall changes were more commonly focal than diffuse, and the duodenum was the most commonly affected region of the gastrointestinal tract for all types of gastrointestinal wall abnormality (abnormal wall layering, abnormal wall thickness, and corrugation), which, given its anatomic proximity to the pancreas, is not surprising. Overall, wall thickening was the most common type of abnormality identified compared with abnormal wall layering or corrugation, and some dogs had ≥1 type of abnormality affecting the same region of the gastrointestinal tract.

Despite limited available literature on this topic, a study evaluating suspected gastric wall edema in dogs with AP noted that 2 of 14 dogs (14.3%) had complete loss of gastric wall layering.^14^ Similarly, our study identified 1 dog with focal complete loss of gastric wall layering, which although often associated with neoplastic disease or ulceration, might be identified in AP secondary to local inflammation or potentially edema. The aforementioned study also reported the submucosa to be the most commonly abnormal individual gastric wall layer, which is in agreement with our findings, where 44.4% of dogs with abnormal gastric wall layering had submucosal involvement.^14^ Another study reported duodenal changes in 18/46 (39.1%) dogs with AP and associated common bile duct obstruction, but did not report the nature of these changes nor the presence of other changes affecting the gastrointestinal tract.^28^ Yet another study reported corrugation and thickening of the duodenal wall alongside gastric wall thickening in 12/38 (31.6%) dogs with AP, but some of those dogs were known to have a previous diagnosis of chronic enteropathy which potentially confounds these results.^15^ Although the latter 2 of these studies did not characterize gastrointestinal wall changes in detail, all of the abnormalities reported also were identified in our population of dogs, which suggests they might be relatively common in dogs with AP.

Only heart rate was an independent predictor of gastrointestinal wall changes in dogs with AP in the multivariable model. There are numerous possible explanations for an increase in heart rate in dogs with AP including pain, fluid losses with resultant hypovolemia through vomiting and diarrhea, nausea, systemic inflammatory response syndrome (SIRS), or myocardial ischemia and thrombosis owing to a hypercoagulable state secondary to AP.^29^ However, it is challenging to explain why heart rate might be related to gastrointestinal wall changes and it is perhaps more likely that heart rate is related to other features of the disease. It is likely that dogs with AP that have increased heart rate are simply sicker and have more severe disease. Although it has been demonstrated in a recent study that severity of ultrasonographic changes in dogs with AP might be correlated with the risk of death, it is yet to be determined whether disease severity or risk of death is correlated with the development of ultrasonographic gastrointestinal wall changes.^8^ A previous study evaluating a clinical severity index, including an organ system severity index, for dogs with AP found a significant association between clinical markers of decreased intestinal integrity, such as functional ileus, regurgitation, melena, hematemesis or anorexia, and nonsurvival.^24^ Although these findings might add weight to the hypothesis that dogs with increasingly severe disease are more likely to have more profound gastrointestinal involvement, this study did not evaluate ultrasonographic appearance of the gastrointestinal wall. In contrast, a recent study incorporating the same organ system severity index did not identify an association between markers of reduced intestinal integrity and death in dogs with AP.^30^ Additional studies to evaluate for correlations between disease severity and gastrointestinal wall changes therefore are needed to explore this relationship further.

Our hypothesis that an increased proinflammatory state as indicated by neutrophilia, neutrophil left shift, and toxic change would be predictive of gastrointestinal wall changes was refuted by our findings although neutrophil toxic change was significantly associated with gastrointestinal wall changes in the univariate model. This hypothesis was based on our assumption that ultrasonographic gastrointestinal wall changes are caused predominantly by extension of local pancreatic inflammation. The lack of association between neutrophil indices and gastrointestinal wall changes in our study might reflect a lack of adequate case numbers to detect such an association, or other limiting factors with the patient population, such as lack of histopathological confirmation of AP for inclusion in the study. It might also reflect a genuine lack of association between neutrophil indices and gastrointestinal wall changes, which would imply that gastrointestinal wall changes in AP result from different mechanisms, or that neutrophil indices are not sensitive markers of pancreatic and gastrointestinal inflammation. It is considered more likely that any pancreatic inflammation present overwhelms other foci of inflammation and thus explains the lack of relationship between gastrointestinal wall changes and neutrophil variables. A recent study evaluating the prognostic value of protease inhibitors and various inflammatory markers in dogs with AP identified lower serum antithrombin activity in nonsurvivors compared with survivors, and higher canine acute pancreatitis severity (CAPS) scores were positively correlated with serum cPLI, C‐reactive protein (CRP) and interleukin‐6 (IL‐6) concentrations and negatively corelated with serum antithrombin activity.^31^ However, this study did not evaluate for relationships between these markers and the presence of gastrointestinal wall changes or ultrasound findings.

Limitations of our study are in part inherent to its retrospective nature. Case evaluation, saving, and storing of ultrasound images and reported ultrasonographic features (such that these would be found under our search terms) were at the discretion of the attending clinician and therefore not standardized. Additionally, clinical record keeping was not standardized, and recording of presenting signs, physical examination findings or ultrasonographic findings might not have been consistent. These factors are particularly pertinent when attempting to exclude cases based upon other comorbidities that could have in themselves altered gastrointestinal wall layering or clinicopathological variables. Furthermore, a full complement of clinical and clinicopathological records was not available for every dog. It is therefore possible that an association between individual variables and the presence of gastrointestinal wall changes might have been missed because of insufficient numbers. In our study, a single board‐certified radiologist reviewed ultrasonographic images. This approach might be considered a limitation because of the partly subjective nature of image interpretation, but the original reports were generated by several board‐certified radiologists and radiology residents under their supervision, which helps to address this limitation to some extent. As discussed, abdominal ultrasonography has variable sensitivity for the diagnosis of AP. Therefore, it is possible that cases where the pancreas appeared normal might have been wrongly categorized as not having AP, and therefore not included in our study. Similarly, the left limb of the pancreas often is difficult to visualize on ultrasonographic examination and therefore cases with disease restricted to the left limb might have been missed and therefore also not included in our study. Additionally, some dogs included in our study had normal serum cPLI (2 of 24 [8.3%] dogs in which cPLI was measured) and were included based upon ultrasonographic findings consistent with pancreatitis, in addition to a cholestatic pattern on serum biochemistry. However, it is recognized that the sensitivity of cPLI is between 70% and 81%, and therefore false‐negative results are possible.^32^ Histopathology was also not available for any of the cases in our study, which ultimately would be required to definitively diagnose AP and to exclude neoplasia as a cause of ultrasonographic pancreatic abnormalities. Additional follow‐up, such as survival information and repeat ultrasonography to demonstrate resolution of gastrointestinal lesions, also would have aided in making this distinction.

In conclusion, ultrasonographic gastrointestinal wall changes were relatively common in this population of dogs with AP and were observed in approximately half of cases. Heart rate was the only clinical or clinicopathological variable independently associated with the presence of gastrointestinal wall changes. However, because of various possible contributing etiologies of tachycardia in this subset of dogs with AP, this finding is considered unlikely to be clinically useful in indicating whether GI wall changes are likely to be present. Our results however may aid clinicians in interpreting ultrasonographic gastrointestinal wall abnormalities in dogs with AP. They highlight that such abnormalities are relatively common, so therefore might be expected, and that even marked changes such as complete loss of GI wall layering might occur in conjunction with AP. However, a repeat ultrasound examination in such cases is recommended to document resolution of GI wall changes and to help exclude other causes of loss of GI wall layering (eg, neoplasia). Our results also provide a basis for further research into the clinical relevance of ultrasonographic GI wall lesions in dogs with AP and the mechanisms by which they occur. In particular, it would be useful to evaluate for a relationship between GI wall changes and increases in inflammatory markers such as CRP. Further work also is needed to evaluate for an association between ultrasonographic GI wall changes, disease severity, and outcome in dogs with AP and to establish whether these changes might have prognostic relevance.

## CONFLICT OF INTEREST DECLARATION

Authors declare no conflict of interest.

## OFF‐LABEL ANTIMICROBIAL DECLARATION

Authors declare no off‐label use of antimicrobials.

## INSTITUTIONAL ANIMAL CARE AND USE COMMITTEE (IACUC) OR OTHER APPROVAL DECLARATION

Approved by the Faculty of Health Sciences, University of Bristol (VIN/20/016).

## HUMAN ETHICS APPROVAL DECLARATION

Authors declare human ethics approval was not needed for this study.
